# Differential profiles of autophagy and Nrf2-mediated antioxidant response in HBeAg-positive versus HBeAg-negative chronic hepatitis B patients

**DOI:** 10.3389/fmicb.2025.1637643

**Published:** 2025-09-16

**Authors:** Pei Liu, Yuna Tang, Jing Wang, Qi An, Ying Zhang, Xuefei Wei, Chao Cui, Yuchen Fan, Huihui Liu, Kai Wang

**Affiliations:** ^1^Department of Hepatology, Qilu Hospital of Shandong University, Jinan, China; ^2^Qilu Hospital of Shandong University Dezhou Hospital, Dezhou, China

**Keywords:** hepatitis B virus, PBMCs, autophagy, oxidative stress, Beclin1, Nrf2-Keap1, p62

## Abstract

**Background:**

HBeAg (hepatitis B e antigen) seroconversion is a key focus in the current treatment of chronic hepatitis B. The processes of autophagy and oxidative stress has been found to be involved in HBV replication and translation. It remains unclear whether this process plays a promotive or inhibitory role in HBeAg seroconversion.

**Methods:**

This study aims to evaluate the role of autophagy in disease progression by quantifying Beclin1 and P62 (Sequestosome 1) as indicators of autophagic activity in hepatitis B patients. Additionally, the Nrf2-Keap1 pathway (nuclear factor erythroid 2-related factor 2/Kelch-like ECH-associated protein 1) serves as a marker of antioxidant defense capacity, thereby allowing an assessment of the contribution of oxidative stress to the progression of the disease in these patients. We examined the expression patterns of these factors in peripheral blood mononuclear cells (PBMCs) and plasma from both HBeAg -positive and HBeAg-negative groups (*n* = 234) using quantitative real-time polymerase chain reaction (RT-qPCR) and ELISA.

**Results:**

The expression levels of Beclin1 and Keap1 were significantly increased compared to those in the HBeAg-negative group (*P* < 0.05). The expression levels of P62, Nrf2, GSH (Glutathione), GSTs (Glutathione S-transferases), and Ho-1 (Heme oxygenase) were significantly decreased compared to those in the HBeAg-negative group (*P* < 0.05). In the HBeAg (+) subgroup stratified by median levels of Beclin1, Nrf2, and Keap1, comparative analysis revealed statistically significant differences in alanine aminotransferase (ALT) expression between Beclin1-high and Beclin1-low groups (*P* < 0.05). Specifically, the Beclin1-high group exhibited significantly higher ALT levels compared to the Beclin1-low group. In the Keap1-stratified analysis, total bilirubin (TBIL), direct bilirubin (DBIL), and indirect bilirubin (IBIL) showed statistically significant differences (*P* < 0.05), with elevated bilirubin levels observed in the Keap1-high group. Further analysis within the HBeAg (+) subgroup demonstrated significant differences in aspartate aminotransferase (AST), DBIL, and alpha-fetoprotein (AFP) expression between Nrf2-high and Nrf2-low groups (*P* < 0.05). The Nrf2-high group displayed higher AST and DBIL levels but lower AFP levels compared to the Nrf2-low group.

**Conclusion:**

Based on our findings, we conclude that autophagy and the Nrf2-Keap1 antioxidant signaling pathway play critical roles in the process of HBeAg seroconversion in chronic hepatitis B patients. Furthermore, monitoring ALT/bilirubin dynamics (linked to autophagy/oxidative stress) may assist in treatment optimization, supporting the quest for clinical cure.

## 1 Introduction

Hepatitis B virus (HBV) infection is currently a global public health problem. According to the World Health Organization, an estimated 296 million people worldwide were chronically infected with HBV in 2019, with about 1.5 million new infections each year (WHO, 2021). In 2019, it was estimated to have killed 820,000 people. According to the Polaris International Epidemiology Collaboration, in 2016, the prevalence of hepatitis B surface antigen (HBsAg) in the general population was 6.1%, and the number of chronic HBV infected patients was 86 million (Polaris Observatory Collaborators, 2018). Despite the widespread availability of effective vaccines and therapy, including immunomodulators (IFN-α-2B and PEG-IFN-α-2A) and reverse transcriptase inhibitors (nucleosides or nucleotide analogues) (Cui et al., 2017), the clinical treatment of chronic hepatitis B still falls short of achieving a satisfactory complete cure. HBV infection continues to be a leading cause of liver cirrhosis and hepatocellular carcinoma. The presence of HBeAg in serum is a marker of active HBV replication and is linked to more severe liver disease. Therefore, in this experiment, we choose to divide the chronic hepatitis B patients into e antigen positive and negative groups, in order to provide early indications for the clinical treatment of chronic hepatitis B.

Autophagy, a cellular homeostatic regulatory system, mediates the degradation of damaged organelles and misfolded proteins via the lysosomal pathway. Its initiation depends on the formation of the class III phosphatidylinositol 3-kinase (class III PI3K) complex mediated by Beclin1 (Liu et al., 2023). HBV bidirectionally regulates autophagy via viral proteins across stages (initiation, autophagosome formation, lysosomal degradation). Moderate autophagy suppresses viral replication via clearance, while excessive mitophagy enhances HBV replication via mitochondrial dysfunction (Lin et al., 2020). Notably, whether hepatitis B e antigen (HBeAg) participates in modulating autophagy homeostasis remains unclear.

During HBV infection, oxidative stress represents another core pathological mechanism. The virus induces reactive oxygen species (ROS) bursts, directly damaging hepatocyte genomic DNA and lipid membrane structures (Jomova et al., 2023; Popa and Popa, 2022; Klieser et al., 2019). The nuclear factor erythroid 2-related factor 2 (Nrf2)-Kelch-like ECH-associated protein 1 (Keap1) pathway constitutes the central regulatory network for cellular antioxidant defense. Under steady-state conditions, Keap1 acts as a negative regulator of Nrf2, maintaining low Nrf2 expression via ubiquitin-proteasomal degradation. Oxidative stress disrupts the Keap1-Nrf2 complex, enabling Nrf2 nuclear translocation to activate the transcription of antioxidant genes, including glutathione (GSH), glutathione S-transferases (GSTs), and heme oxygenase-1 (HO-1). Importantly, autophagy interacts with the Nrf2 pathway through molecular crosstalk: the autophagy receptor protein p62/SQSTM1 selectively binds Keap1 via its Keap1-interacting region (KIR) domain, targeting Keap1 for co-degradation with autophagosomes (Thiruvengadam et al., 2023; Bender and Hildt, 2019; Liu et al., 2015; Miyakawa et al., 2022). This process relieves Keap1-mediated inhibition of Nrf2, establishing a positive feedback regulatory loop.

Based on these mechanisms, this study enrolled HBeAg-positive and HBeAg-negative chronic hepatitis B (CHB) patients. Autophagy levels were assessed by measuring the expression of key markers (Beclin1 and p62) via RT-qPCR. The antioxidant capacity of the Nrf2-Keap1 pathway was evaluated by quantifying Nrf2 and Keap1 expression (RT-qPCR) and downstream antioxidant enzymes (GSH, GSTs, HO-1) via ELISA. Group comparisons and correlation analyses were performed to explore the relationship between HBeAg status and autophagy-oxidative stress dynamics. The scientific significance of this study lies in elucidating whether autophagy-oxidative stress can play a role in the serological conversion of HBeAg, thereby providing theoretical foundations for personalized therapeutic strategies targeting autophagy dynamics modulation or Nrf2 pathway activation. This study utilized peripheral blood mononuclear cells (PBMCs), leveraging their non-invasive and easily accessible characteristics to serve as a surrogate tissue for liver samples in the investigation within this specific experimental context. Standardized techniques facilitated clinical translation. However, limitations include their inability to accurately mirror intrahepatic pathology, inherent cellular heterogeneity, and potential *ex vivo* stress artifacts. Future research should integrate hepatocyte models and mechanistic experiments to validate the underlying pathways.

## 2 Materials and methods

### 2.1 Study population

**FIGURE 1:**
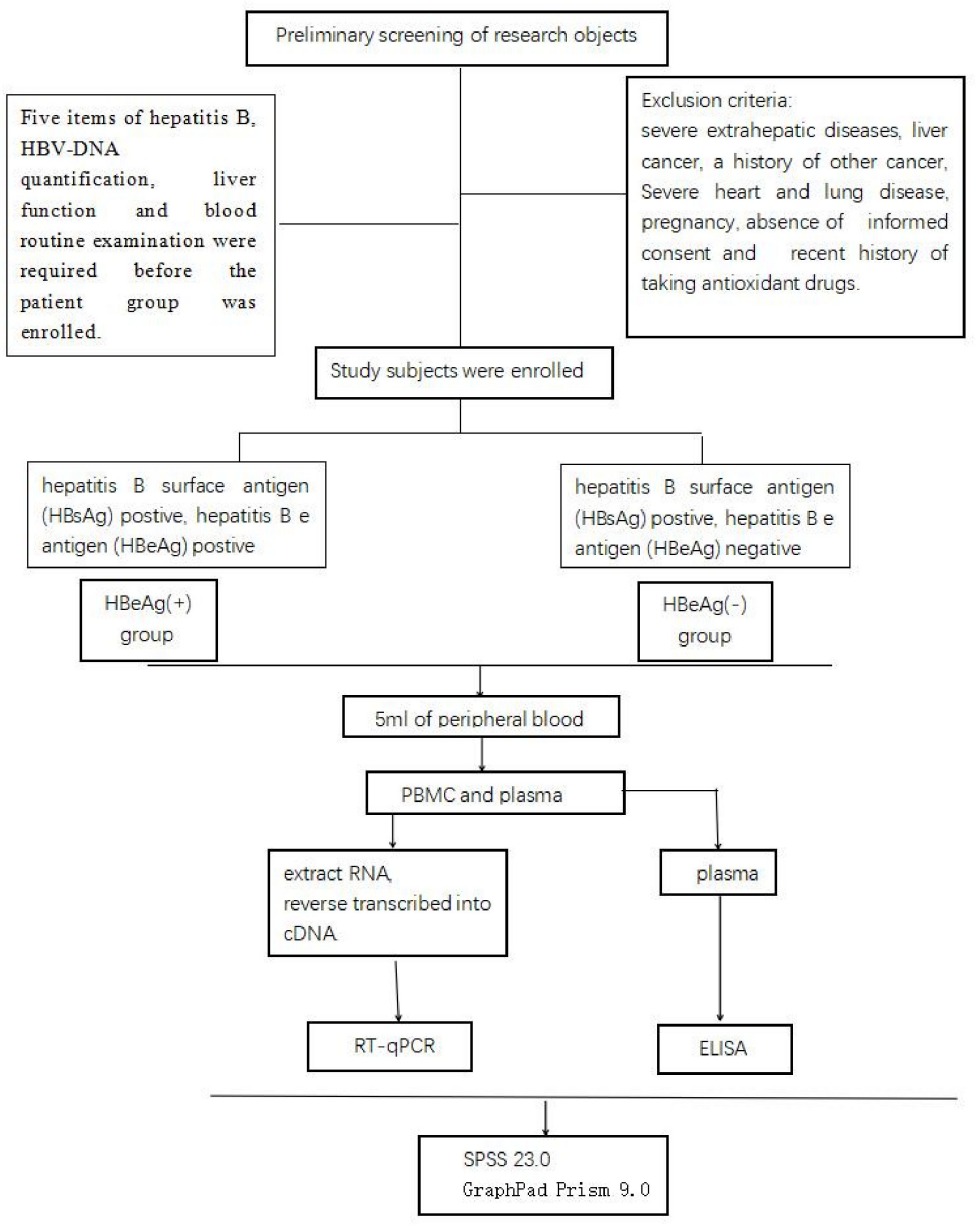
Process of research.

The hepatitis B patients in the Department of Hepatology, Qilu hospital of Shandong University, were collected and divided into HBeAg (+) group, and HBeAg (−) group. Five items of hepatitis B testing, HBV-DNA quantification, liver function tests, and blood routine examination were required before the patient groups were enrolled. The inclusion criteria for CHB patients were determined in accordance with the updated CHB management practice guidelines of the World Health Organization (WHO) in 2022. The inclusion criteria were as follows: (1) Aged between 18 and 60 years; (2) Patients with impaired liver function were excluded; (3) HBsAg seropositive status confirmed for ≥6 months. The exclusion criteria included severe extrahepatic diseases, liver cancer, a history of other cancer, severe heart and lung disease, pregnancy, absence of informed consent, and a recent history of taking antioxidant drugs. The HBeAg (+) group were diagnosed based on hepatitis B surface antigen (HBsAg) positive and hepatitis B e antigen (HBeAg) postive. The HBeAg (−) group were diagnosed based on hepatitis B surface antigen (HBsAg) postive and hepatitis B e antigen (HBeAg) negative. Finally, a total of 97 HBsAg (+) patients and 137 HBsAg (−) patients were included (Figure 1). This study has been reviewed by the Research Ethics Committee of Qilu Hospital of Shandong University (Ethics approval number: KYLL-202306-021-1).

### 2.2 Serum collection and PBMCs isolation

Five milliliters of peripheral blood was collected using vacuum vessels. PBMCs and plasma were extracted from the samples in the Hepatology Department’s laboratory at Shandong University Qilu Hospital. The Trizol method was employed to extract RNA from PBMCs, which was then reverse transcribed into cDNA and stored at −80 °C in the refrigerator for future use (Figure 1).

### 2.3 Measurement of expression levels

Real-time fluorescence quantitative polymerase chain reaction (RT-qPCR) was used to detect the expression levels of Beclin1, P62, Keap1, and Nrf2. (Primer sequences: P62F, GACTACGACTTGTGTAGCGTC; P62R: AGTGTCCGTGTTT CACCTTCC. Beclin1F: CCATGCAGGTGAGCTTCGT; Beclin1R: GAATCTGCGAGAGACACCATC. Nrf2F: TTCCCGGTCACATC GAGAG; Nrf2R: TCCTGTTGCATACCGTCTAAATC. Keap1F: CTGGAGGATCATACCAAGCAGG; Keap1R: GGATACCCTC AATGGACACCAC). The expression of antioxidant enzymes (HO-1, GSH, and GSTs) was detected by ELISA (the kit was obtained from Langton) (Figure 1).

### 2.4 Statistical analysis

SPSS 23.0 software and GraphPad Prism 9.0 software were used for statistical analysis. The demographic characteristics are presented as medians (ranges). Clinical indicators and experimental detection indicators of subjects in each group were analyzed. Expression levels of factors were calculated using RT-qPCR data, and expression levels of enzymes were calculated using the Logistic regression fitting curve in ELisacalc software. Mann–Whitney U test and Spearman correlation analysis were used for comparison between groups, and *P* < 0.05 was statistically significant (Figure 1).

## 3 Results

### 3.1 Comparison of clinical data between HBeAg (+) and HBeAg (−) groups

A general analysis of clinical characteristics was conducted on 234 patients. The expression levels of clinical indicators including ALT (Alanine Aminotransferase), AST (Aspartate Aminotransferase), GDH (Glutamate Dehydrogenase), ADA (Adenosine Deaminase), bilirubin, and AFP (Alpha-Fetoprotein) were compared between HBeAg-positive and HBeAg-negative groups, with all clinical parameters described as median (range). The cohort comprised 149 male patients and 85 female patients, with enrolled patients aged 18–60 years. The median age was 50 years in the HBeAg-positive group compared to 43 years in the HBeAg-negative group. Detailed clinical data are presented in Table 1.

**TABLE 1:** The clinical characteristics of HBeAg patients and HBeAb patients.

	HBeAg (+) (*n* = 97)	HBeAg (−) (*n* = 137)
Sex (male/female)	63/34	86/51
Age (years)	50 (18–60)	43 (18–60)
ALT (U/L)	24 (8–56)	20 (5.1–54)
AST (U/L)	24 (10–40)	22 (12–48)
GDH (U/L)	4.3 (1.7–8)	3.6 (1–7.3)
ADA (U/L)	13 (6–31)	13 (7–20)
TBIL (umol/l)	10.9 (3.5–20.7)	11.1 (2.8–19)
DBIL (umol/l)	3.65 (1.5–6.6)	3.95 (1.4–6.8)
IBIL (umol/l)	6.8 (2–11.8)	7.15 (1.3–12)
AFP (ng/ml)	2.75 (1.12–8.91)	2.33 (0.91–7.79)

ADA, adenosine deaminase; AFP, α-fetoprotein; ALT, alanine aminotransferase; AST, aspartate aminotransferase; DBIL, direct bilirubin; GDH, glutamic dehydrogenase; IBIL, indirect bilirubin; TBIL, total bilirubin. Between-group comparisons of continuous variables were performed using the Mann–Whitney U test (for non-normally distributed data), with values expressed as median. ****P < 0.0001, ***p < 0.001, **p < 0.01, *p < 0.05.

### 3.2 The expression level of factors in CHB patients

**FIGURE 2:**
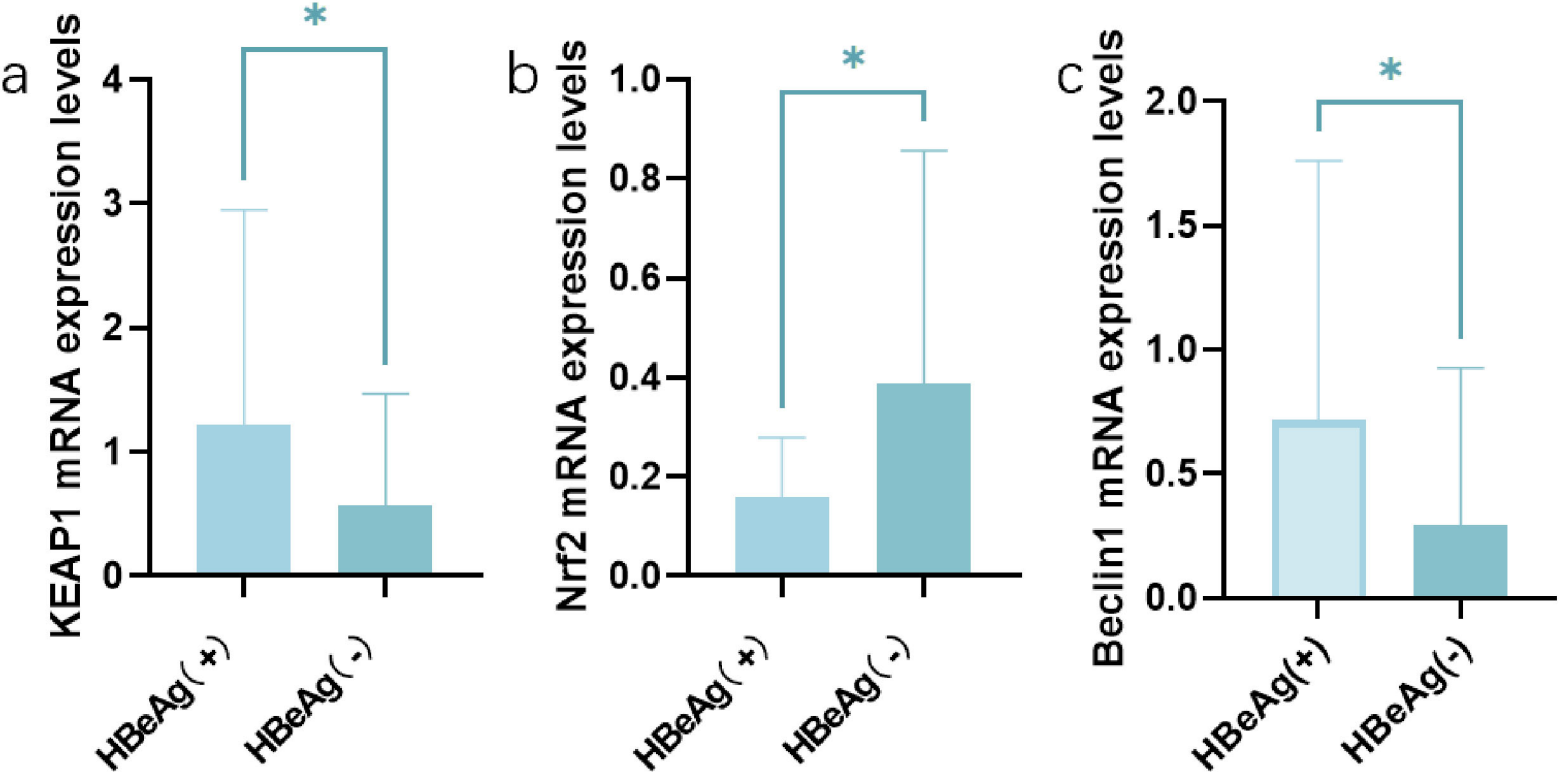
The expression patterns of Keap1, Nrf2, and Beclin1 in HBeAg (+) and HBeAg (–) group. **(a)** Keap1. **(b)** Nrf2. **(c)** Beclin1. *P < 0.05, data compared using the Mann–Whitney U test between HBeAg (+) (*n* = 97) and HBeAg (–) (*n* = 137) patient groups.

Quantitative real-time polymerase chain reaction was performed on the cDNA of patients in the HBeAg (+) group and the HBeAg (−) group. The observed transcriptional upregulation of Keap1 and downregulation of Nrf2 (*P* < 0.05) may reflect a compensatory response to oxidative stress. While the canonical NRF2-Keap1 pathway primarily regulates NRF2 protein stability through Keap1-mediated ubiquitination (Bender and Hildt, 2019; Liu et al., 2015), the transcriptional changes in our study suggest a potential feedback loop or upstream regulatory mechanisms modulating their mRNA expression in CHB progression (Figure 2).

### 3.3 The expression level of enzymes in CHB patients

**FIGURE 3:**
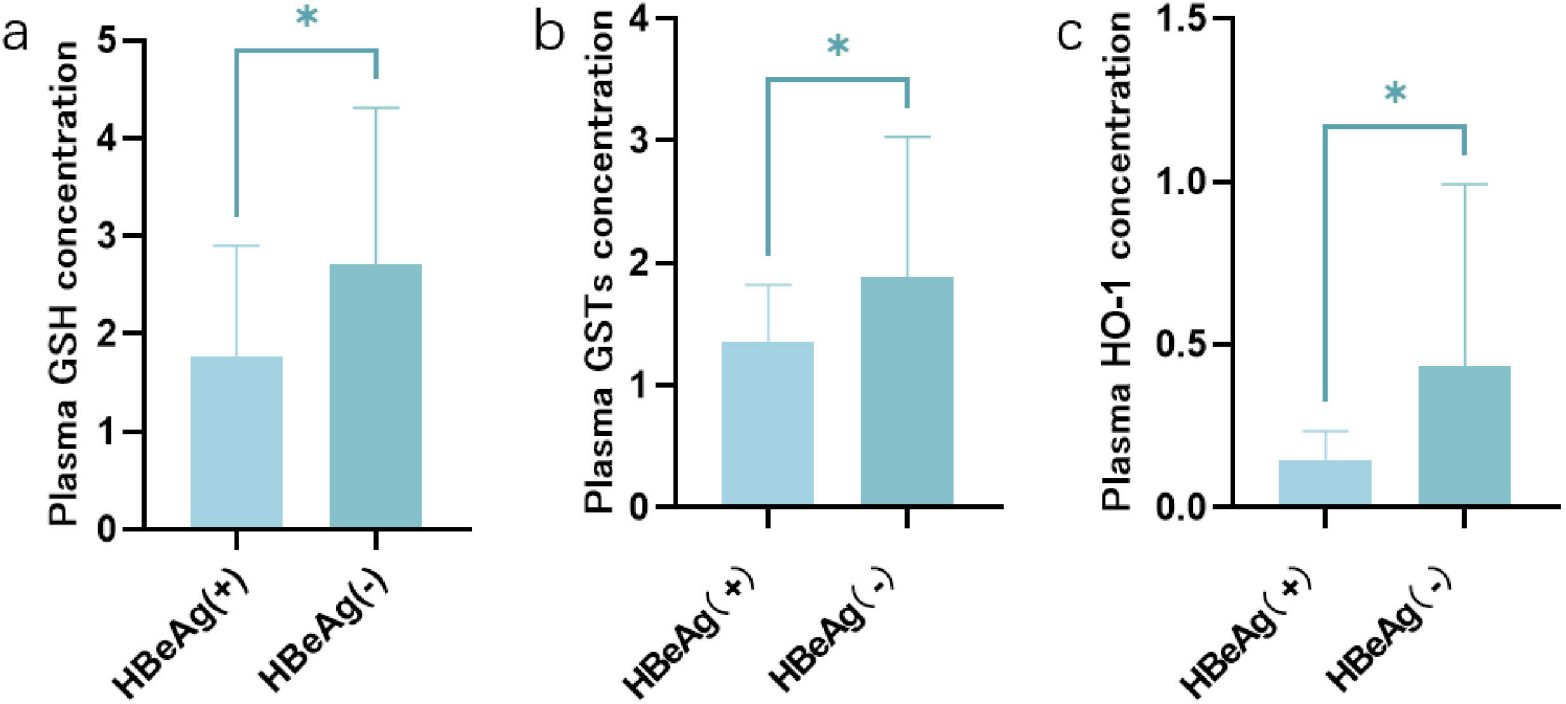
The expression patterns of GSH, GSTs, and Ho-1 in HBeAg (+) and HBeAg (–) group. **(a)** GSH. **(b)** GSTs. **(c)** HO-1. *P < 0.05, data compared using the Mann–Whitney U test.

Enzyme-linked immunosorbent assay (ELISA) was performed on the serum of patients in the HBeAg (+) group and HBeAg (−) group. Compared with the HBeAg (−) group, the expression levels of HO-1, GSH and GSTs in the HBeAg (+) group were lower than those in the HBeAg (−) group (*P* < 0.05) (Figure 3).

### 3.4 The expression levels of factors and enzymes in CHB patients suggest a relationship between autophagy and Nrf2 and Keap1

**FIGURE 4:**
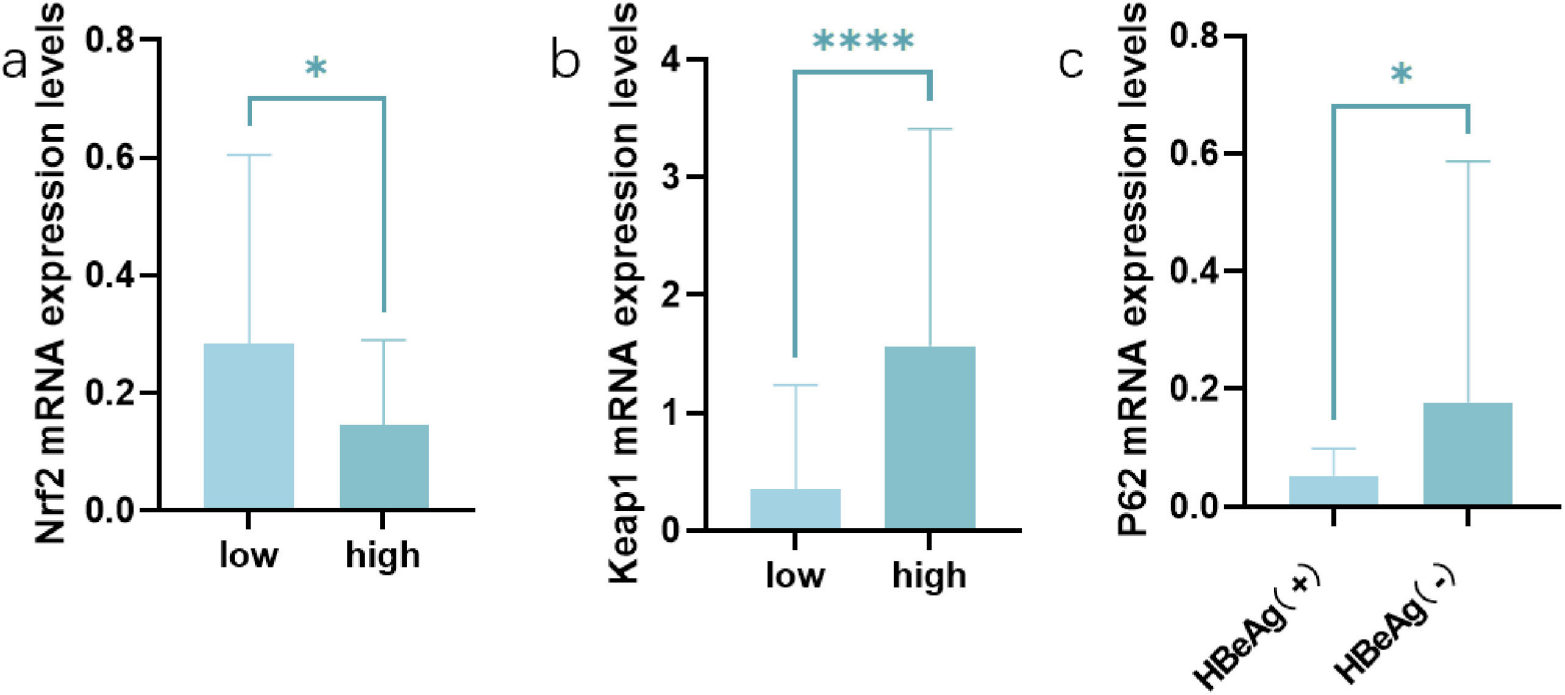
The expression patterns of Nrf2, Keap1 and P62 in different groups. **(a)** The expression patterns of Nrf2 in Beclin1 increase group and Beclin1 decrease group. **(b)** The expression patterns of Keap1 in Beclin1 increase group and Beclin1 decrease group. **(c)** The expression patterns of P62 in HBeAg (+) and HBeAg (–) group. ****P < 0.0001,*p < 0.05, data compared using the Mann–Whitney U test.

**FIGURE 5:**
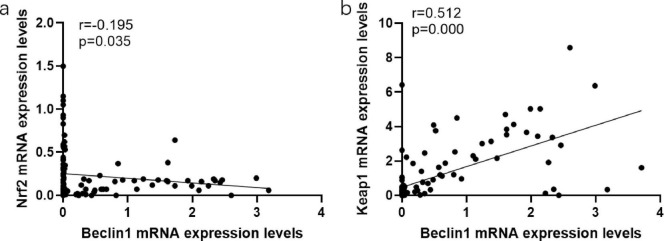
The associations between the expression levels of oxidative stress characteristics and autophagy characteristics. **(a)** The associations between the expression levels of Nrf2 and Beclin1. **(b)** The associations between the expression levels of Keap1 and Beclin1. Data compared using the Spearman correlation analysis.

Compared with HBeAg (−) group, P62 expression in the peripheral blood of HBeAg (+) group was lower (*P* < 0.05). And the HBeAg (+) group and HBeAg (−) group were divided into the Beclin1 increase group and Beclin1 decrease group, with the median Beclin1 level as the baseline. We found that in the Beclin1 increased group, Nrf2 levels were lower than those in the Beclin1-decreased group, and Keap1 levels were higher than those in the Beclin1-decreased group (*P* < 0.05) (Figure 4). Furthermore, we used Spearman correlation analysis and concluded that Beclin1 is negatively correlated with Nrf2 and positively correlated with Keap1 (Figure 5).

### 3.5 The expression levels of autophagy-related factor correlates with HBV-DNA levels in chronic hepatitis B patients

**FIGURE 6:**
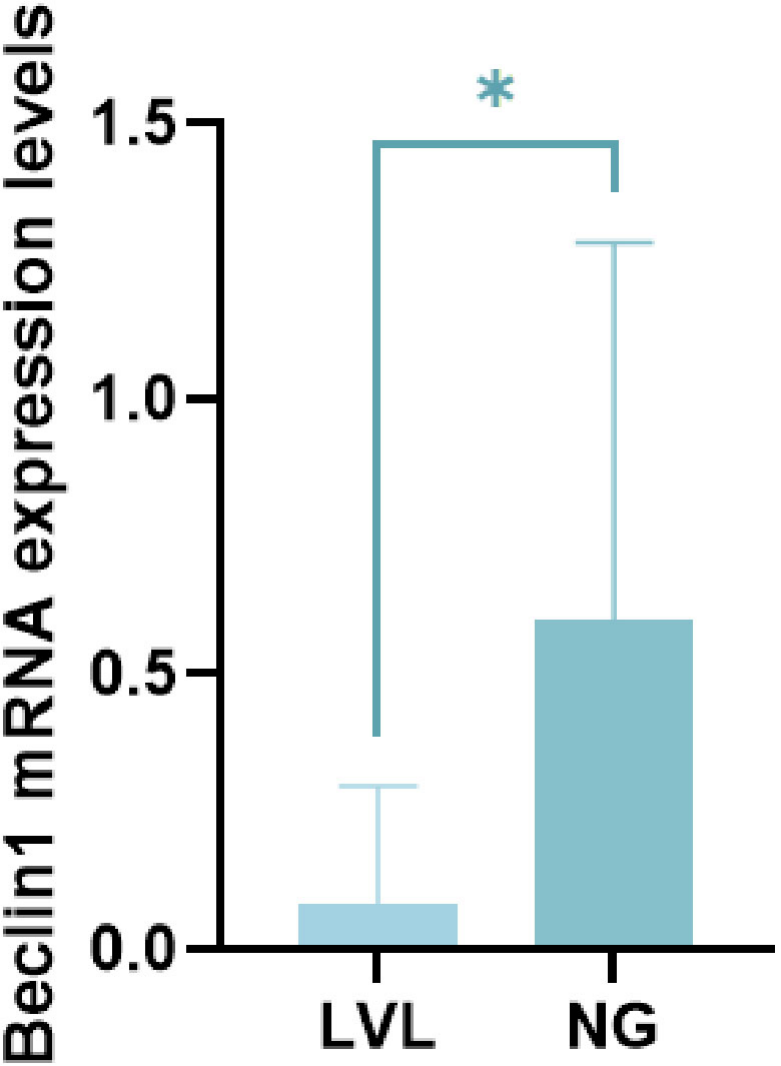
The Beclin1 expression level in LVL group and normal group. *P < 0.05, data compared using the Mann–Whitney U test.

The serological conversion of e antigen often indicates a decline in viral replication ability. Currently, in clinical treatment, there exists a stage of LVL/LLV in the disease course. LVL is defined as the serum HBV DNA level being above the lower limit of detection (50 IU/ml) but less than 2,000 IU/ml, after excluding potential testing errors. In patients receiving antiviral treatment, LVL is commonly referred to as low-level viremia (LLV) (Hao et al., 2024). The NG (Non-Low Viral Load) group comprises chronic HBV-infected patients with serum HBV-DNA levels exceeding the threshold of the LVL (Low Viral Load) group (HBV-DNA ≥ 2,000 IU/mL). Therefore, we analyzed the level of autophagy in patients during the LVL/LLV stage. Compared with LVL group, Beclin1 expression in peripheral blood of NG was lower (*P* < 0.05) (Figure 6).

### 3.6 Associations between the Nrf2, Keap1, and Beclin1 expression levels and clinical features in CHB patients

Patients were stratified into HBeAg-positive and HBeAg-negative groups. Within each cohort, subgroups were established based on the median Beclin1 expression level (Beclin1-high vs. Beclin1-low subgroups) for comparative analysis of clinical parameters. In the HBeAg-positive cohort, comparative analysis of hepatic function markers revealed: Alanine aminotransferase (ALT) demonstrated statistically significant elevation (*P* < 0.05) in the Beclin1-high subgroup compared to the Beclin1-low subgroup (Table 2). Subsequent stratification by Nrf2/Keap1 signaling axis mediators (dichotomized at median levels) yielded additional findings: Keap1-elevated subgroup exhibited significantly higher total bilirubin, direct bilirubin, and indirect bilirubin compared to Keap1-reduced counterparts (all *P* < 0.05) (Table 3a). In HBeAg-positive patients with Nrf2 overexpression: Elevated AST and DBIL. Reduced AFP levels relative to Nrf2-low subgroup (*P* < 0.05 for all comparisons) (Table 3b).

**TABLE 2:** Characteristic differences between the high Beclin1 expression group and the low Beclin1 expression group.

	HBeAg (+) (*n* = 68)		*P-*value	HBeAg (−) (*n* = 56)		*P*-value
	Low Beclin1 expression group	High Beclin1 expression group		Low Beclin1 expression group	High Beclin1 expression group	
ALT (U/L)	23 (9–37)	31 (13–56)	0.007**	22.5 (5.1–39)	21 (11–43)	0.142
AST (U/L)	22 (15–32)	25 (14–36)	0.105	26 (12–40)	22.5 (13–37)	0.35
GDH (U/L)	4.1 (1.8–7.9)	5.05 (2.8–7.3)	1	4.4 (1.2–7)	3.85 (1.7–6.2)	0.374
ADA (U/L)	15.5 (6–19)	14 (11–19)	0.929	14 (7–20)	14 (6–18)	0.93
TBIL (umol/l)	8.6 (5.3–18.6)	10.35 (4.7–18.3)	0.35	12.7 (2.8–18.8)	12.35 (4.7–19)	0.7
DBIL (umol/l)	3 (2.2–5.4)	4.05 (1.7–6.6)	0.062	4.25 (1.4–6.2)	4.15 (1.7–6.7)	0.974
IBIL (umol/l)	5.5 (3–9.1)	6.7 (3–11.4)	0.461	8.4 (1.3–12)	7.65 (2.9–10.4)	0.706
AFP (ng/ml)	2.6 (1.5–5.79)	2.88 (1.12–4.01)	0.767	2.35 (1.01–8.91)	3.03 (1.14–5.52)	0.711

ADA, adenosine deaminase; AFP, α-fetoprotein; ALT, alanine aminotransferase; AST, aspartate aminotransferase; DBIL, direct bilirubin; GDH, glutamic dehydrogenase; IBIL, indirect bilirubin; TBIL, total bilirubin. Between-group comparisons of continuous variables were performed using the Mann–Whitney U test (for non-normally distributed data), with values expressed as median.

****P < 0.0001, ***p < 0.001, **p < 0.01, *p < 0.05.

**TABLE 3a:** Characteristic differences between the high Keap1 expression group and the low Keap1 expression group.

	HBeAg (+) (*n* = 40)		*P*-value	HBeAg (−) (*n* = 62)		*P*-value
	Low Keap1 expression group	High Keap1 expression group		Low Keap1 expression group	High Keap1 expression group	
ALT (U/L)	23 (9–37)	26.5 (13–56)	0.149	20 (5.1–39)	22 (8–54)	0.622
AST (U/L)	21 (14–32)	24.5 (17–36)	0.081	22 (13–40)	26 (12–37)	0.627
GDH (U/L)	3.95 (1.8–7.9)	4.15 (2.8–7.3)	0.933	3.95 (1.7–6.2)	6.75 (1.2–7)	0.118
ADA (U/L)	15 (6–19)	16 (11–19)	0.518	14 (10–15)	18 (7–20)	0.588
TBIL (umol/l)	8.4 (4.7–16.9)	11.7 (7.6–18.6)	0.024*	10.3 (2.8–18.8)	15 (4.8–19)	0.005**
DBIL (umol/l)	2.95 (1.7–4.8)	4.1 (2.8–6.6)	0.01*	3.6 (1.4–6.7)	5.3 (2.9–6.7)	0.003**
IBIL (umol/l)	5.4 (3–9.8)	7.1 (4.3–11.4)	0.02*	6.4 (1.3–12)	10 (4.7–10.4)	0.002**
AFP (ng/ml)	2.95 (1.5–5.79)	2.73 (1.12–5.91)	0.988	2.39 (0.91–8.91)	2.39 (1.14–6.09)	0.894

ADA, adenosine deaminase; AFP, α-fetoprotein; ALT, alanine aminotransferase; AST, aspartate aminotransferase; DBIL, direct bilirubin; GDH, glutamic dehydrogenase; IBIL, indirect bilirubin; TBIL, total bilirubin. Between-group comparisons of continuous variables were performed using the Mann–Whitney U test (for non-normally distributed data), with values expressed as median.

****P < 0.0001, ***p < 0.001, **p < 0.01, *p < 0.05.

**TABLE 3b:** Characteristic differences between the high Nrf2 expression group and the low Nrf2 expression group.

	HBeAg (+) (*n* = 66)		*P*-value	HBeAg (−) (*n* = 56)		*P*-value
	Low Nrf2 expression group	High Nrf2 expression group		Low Nrf2 expression group	High Nrf2 expression group	
ALT (U/L)	23 (9–56)	26.5 (13–48)	0.19	19.5 (5.1–37)	23 (9–54)	0.325
AST (U/L)	21 (14–27)	24.5 (16–36)	0.021*	21.5 (14–40)	26 (14–38)	0.799
GDH (U/L)	3.55 (1.8–4.8)	5.7 (2.7–7.9)	0.121	3.95 (1.7–5.1)	7 (6.5–7)	0.059
ADA (U/L)	11.5 (6–16)	15 (9–19)	0.055	14 (6–15)	14 (13–20)	0.467
TBIL (umol/l)	8.5 (4.7–18.6)	10.2 (7.6–17.6)	0.092	12 (3.4–18.8)	10.7 (7.7–18.7)	0.768
DBIL (umol/l)	2.9 (1.7–6.6)	3.6 (2.7–6)	0.009**	4.15 (1.9–6.7)	4.1 (2.9–6.7)	0.376
IBIL (umol/l)	5.6 (3–9.8)	6.8 (4.3–11.4)	0.183	7.55 (1.4–10.6)	6.85 (4.8–12)	0.561
AFP (ng/ml)	3.14 (1.61–5.91)	2.53 (1.12–5.79)	0.006**	2.875 (1.05–8.91)	2.2 (1.6–6.09)	0.326

ADA, adenosine deaminase; AFP, α-fetoprotein; ALT, alanine aminotransferase; AST, aspartate aminotransferase; DBIL, direct bilirubin; GDH, glutamic dehydrogenase; IBIL, indirect bilirubin; TBIL, total bilirubin. Between-group comparisons of continuous variables were performed using the Mann–Whitney U test (for non-normally distributed data), with values expressed as median.

****P < 0.0001, ***p < 0.001, **p < 0.01, *p < 0.05.

## 4 Discussion

Autophagy and oxidative stress can exist in the progression of inflammation and fibrosis. The above processes must exist during the development and progression of hepatitis B to cirrhosis and Hepatic carcinoma (Lin et al., 2020; Zhang, 2020; Jiang et al., 2021; Xie et al., 2018; Sartorius et al., 2020). It is well-known that the important step in the course of hepatitis B cure is the negative conversion of e antigen. Therefore, in this study, hepatitis B patients were divided into HBeAg (+) and HBeAg (−) groups, and the levels of autophagy and oxidative stress in peripheral blood mononuclear cells (PBMCs) and plasma were analyzed respectively. In this study, it can be found that HBeAg (+) patients have the ability to activate autophagy, but their antioxidant capacity is decreased, and the downstream expression enzyme is also decreased. And we also analyzed the level of autophagy in patients during the LVL/LLV stage and the normal group (Hao et al., 2024). Compared with LVL group, Beclin1 expression in peripheral blood of normal group was lower. Clinically, autophagy was found to have a greater impact on ALT, whereas oxidative stress had a more pronounced effect on bilirubin levels.

In our study, P62 expression in peripheral blood of HBeAg (+) group was lower compared with that of the HBeAg (−) group. Furthermore, we concluded that Beclin1 is negatively correlated with Nrf2 and positively correlated with Keap1. In other researches, they found Keap1 binds to the Neh2 domain of Nrf2 through its Kelch domain, inhibiting Nrf2’s transcriptional activity and promoting its degradation. During oxidative stress, Keap1 undergoes a conformational change, leading to the release of Nrf2 from the Keap1 complex. Nrf2 then translocates to the nucleus and activates downstream antioxidant genes. Also, P62 binds to Keap1 through its PB1 domain, disrupting the interaction between Keap1 and Nrf2, facilitating the accumulation of Nrf2 and activating antioxidant responses. Through our research, this mechanism may also works.

In liver diseases such as NASH, autophagy levels in the liver tissue of NASH patients are reduced, leading to the worsening of many characteristics of NAFLD, such as fat droplet accumulation, increased damage to hepatocytes, and elevated endoplasmic reticulum stress (Ren et al., 2024). During liver fibrosis, a key marker of the activation of hepatic stellate cells (HSCs) is the disappearance of fat droplets. The digestion of fat droplets by autophagy is considered an energy source driving HSC activation. Studies have found that inhibiting autophagy can downregulate the fibrotic state of HSCs. Autophagy also inhibits fibrosis, as it has been reported that autophagy-mediated miR-30a-5p/ATG5 axis can alleviate liver fibrosis through induced apoptosis suppression. A large body of evidence suggests that the mechanisms underlying the pathogenesis of alcoholic liver disease (ALD) are associated with alcohol-induced abnormal autophagy. In cancer research, autophagy has a dual role in promoting and inhibiting tumors. Autophagy can suppress tumor growth in the early stages of tumorigenesis; however, it can also provide energy to starving tumor cells. Autophagy can lead to multidrug resistance in tumor cells thereby reducing their sensitivity to radiation. In current research on autophagy and oxidative stress, during acute events, autophagy or its regulation of inflammatory signals can prevent organs from developing severe inflammatory responses, while excessive activation of autophagy can have the opposite effect (Ma et al., 2024; Shen et al., 2024). In chronic diseases, such as COPD and liver fibrosis, increased autophagy levels have been observed, contributing to disease progression (Ren et al., 2024; Lin et al., 2024). In inflammation-related autoimmune diseases, such as UC and other gastrointestinal diseases (Kim et al., 2012), numerous studies have focused on autophagy and oxidative stress. In the occurrence and development of tumors, autophagy also plays a role in promoting or inhibiting tumor progression and in drug resistance.

While our study highlights the clinical correlations between Beclin-1, the Nrf2-Keap1 pathway, and HBeAg seroconversion in chronic hepatitis B (CHB), it is critical to contextualize these findings within the intricate interplay of HBV pathogenesis, autophagy, and oxidative stress. On one hand, (HBx) has been shown to hijack the autophagic machinery by activating the AMPK/mTOR axis to promote viral envelopment and secretion, while simultaneously suppressing autophagic degradation through ubiquitination-mediated destabilization of autophagy receptors (e.g., p62/SQSTM1) (Wang et al., 2020; Kim et al., 2022). On the other hand, Beclin-1 upregulation during HBeAg seroconversion might represent a compensatory mechanism to counteract viral-induced ER (endoplasmic reticulum) stress, as HBV X protein (HBx) disrupts mitochondrial bioenergetics and increases ROS (reactive oxygen species) production, further exacerbating hepatocyte vulnerability to inflammatory damage. During HBeAg seroconversion, the upregulation of Beclin-1 may represent a compensatory mechanism to counteract virus-induced ER stress. This ER stress is exacerbated by HBx-mediated disruption of mitochondrial bioenergetic metabolism and increased ROS production, thereby heightening hepatocyte susceptibility to inflammatory injury. Current studies have demonstrated that HBV can hijack the host hypoxia-inducible factor (HIF) signaling pathway through evolutionarily conserved hypoxia-responsive elements (HREs), leveraging the hypoxic microenvironment of the liver to enhance its own transcription and replication. Concurrently, the hypoxic microenvironment may promote viral persistence and liver injury in chronic hepatitis B (CHB) via HIF-mediated pathways (Wing et al., 2021). P62, a key adaptor protein, has been implicated in mitochondrial ubiquitination. Emerging evidence highlights the dual role of the ubiquitination system in HBV-induced hepatocellular carcinoma (HCC). While ubiquitination contributes to host antiviral defense mechanisms, it is also exploited by the virus to facilitate viral replication and oncogenesis (Yamada et al., 2019). This “double-edged sword” characteristic underscores the complex interplay between the ubiquitin-proteasome system and HBV pathogenesis, balancing protective responses against viral infection with inadvertent promotion of viral survival and carcinogenesis (Kar et al., 2024). HBx, a multifunctional viral regulator, interacts directly with the mitochondrial outer membrane, compromising its structural integrity (Zhong et al., 2015, 2017; Zhou et al., 2016). This interaction is mediated through HBx binding to voltage-dependent anion channel 3 (VDAC3), a key component of the permeability transition pore (PTP) (Lee et al., 2018), which disrupts PTP function by elevating cytoplasmic and mitochondrial Ca^2+^ concentrations (Reina et al., 2016). These events culminate in mitochondrial membrane potential collapse and excessive ROS generation. Notably, both HBx and large hepatitis B surface protein (LHBs) act as transcriptional co-activators that induce c-Raf phosphorylation, a prerequisite for HBV-dependent Nrf2 activation (Kalantari et al., 2023). Activated Nrf2 translocates to the nucleus and binds antioxidant response element (ARE) sequences, upregulating cytoprotective genes such as NAD(P)H quinone oxidoreductase 1 (NQO1) and heme oxygenase-1 (HO-1). And according to the analysis, it can be roughly estimated that autophagy has a greater effect on ALT, while oxidative stress has a greater effect on bilirubin. This point, which has been overlooked in recent studies, is the highlight of this study. Thus, in the future clinical work, early detection of clinical indicators in patients with chronic hepatitis B, especially for patients with abnormal ALT and bilirubin levels. Specific measurement of patients’ peripheral blood autophagy and antioxidant indicators should be conducted to guide specific medication for patients. Our findings suggest that Beclin-1 and Nrf2-Keap1 pathway dysregulation during HBeAg seroconversion reflect HBV’s subversion of autophagy and oxidative stress networks to sustain hepatic inflammation and metabolic dysfunction. Future studies should explore spatial multi-omics approaches to map autophagy-ROS crosstalk in HBV-infected liver niches, alongside preclinical validation of pathway-specific therapies to improve CHB outcomes.

Although there are previous studies on autophagy and oxidative stress in hepatitis B virus infection, the samples were mostly collected from liver cancer patients caused by hepatitis B. However, this study is relatively novel in reflecting autophagy and oxidative stress abilities through peripheral blood related indicators in hepatitis B patients. Nevertheless, there are defects in this experiment: PBMCs cannot completely simulate the intracellular environment. Moreover, because hepatitis B patients rarely undergo liver biopsy, there is a lack of samples. However, according to the research results of related articles, autophagy is also associated with immune cells within PBMCs (Son et al., 2021; Yoon et al., 2023; Geertsema et al., 2023; Ren et al., 2024; Chandrasekaran et al., 2023; Debnath et al., 2023). Yet, this experiment did not subdivide each immune cell group in PBMCs. This study focused on the early phase of HBeAg seroconversion (defined as HBeAg loss with anti-HBe emergence), aiming to characterize autophagy and oxidative stress dynamics. HBeAg seroconversion primarily reflects immune transition, whereas HBsAg/HBV-DNA changes (e.g., HBsAg clearance) are delayed events. Thus, our design prioritized molecular mechanisms over long-term virological correlations. We plan to conduct a future study investigating the regulatory effects of HBV-DNA load on autophagy/oxidative stress pathways and exploring the association between HBsAg kinetics and immune microenvironment remodeling post-seroconversion. In addition, this study is single-center and lacks a verification set, and the number of subjects included is not large, which may lead to errors. An important limitation is the lack of systematic assessment of antiviral medication use among participants. As these agents may directly influence oxidative stress and autophagy pathways–key mechanisms under investigation–the absence of medication screening constitutes a potential confounding factor in our analysis.

## Data Availability

The raw data supporting the conclusions of this article will be made available by the authors, without undue reservation.

## References

[B1] Bender D. Hildt E. (2019). Effect of hepatitis viruses on the Nrf2/Keap1-signaling pathway and its impact on viral replication and pathogenesis. *Int. J. Mol. Sci.* 20:4659. 10.3390/ijms20184659 31546975 PMC6769940

[B2] Chandrasekaran V. Hediyal T. Anand N. Kendaganna P. Gorantla V. Mahalakshmi A. (2023). Polyphenols, autophagy and neurodegenerative diseases: A review. *Biomolecules* 13:1196. 10.3390/biom13081196 37627261 PMC10452370

[B3] Cui F. Shen L. Li L. Wang H. Wang F. Bi S. (2017). Prevention of chronic hepatitis b after 3 decades of escalating vaccination policy, China. *Emerg. Infect. Dis.* 23 765–772. 10.3201/eid2305.161477 28418296 PMC5403029

[B4] Debnath J. Gammoh N. Ryan K. (2023). Autophagy and autophagy-related pathways in cancer. *Nat. Rev. Mol. Cell Biol.* 24 560–575. 10.1038/s41580-023-00585-z 36864290 PMC9980873

[B5] Geertsema S. Bourgonje A. Fagundes R. Gacesa R. Weersma R. van Goor H. (2023). The NRF2/Keap1 pathway as a therapeutic target in inflammatory bowel disease. *Trends Mol. Med.* 29 830–842. 10.1016/j.molmed.2023.07.008 37558549

[B6] Hao K. Dong Y. Fan Y. Jiang X. Xiong X. Gao L. (2024). Analysis of the incidence of low viral load/low-level viremia and its associated factors in patients with HBV-related primary liver cancer. *Chin. J. Hepatol.* 32 910–915. 10.3760/cma.j.cn501113-20230829-00076 39528326 PMC12898940

[B7] Jiang Y. Han Q. Zhao H. Zhang J. (2021). The mechanisms of HBV-induced hepatocellular carcinoma. *J. Hepatocell. Carcinoma* 8 435–450. 10.2147/JHC.S307962 34046368 PMC8147889

[B8] Jomova K. Raptova R. Alomar S. Alwasel S. Nepovimova E. Kuca K. (2023). Reactive oxygen species, toxicity, oxidative stress, and antioxidants: Chronic diseases and aging. *Arch. Toxicol.* 97 2499–2574. 10.1007/s00204-023-03562-9 37597078 PMC10475008

[B9] Kalantari L. Ghotbabadi Z. Gholipour A. Ehymayed H. Najafiyan B. Amirlou P. (2023). A state-of-the-art review on the NRF2 in Hepatitis virus-associated liver cancer. *Cell Commun. Signal.* 21:318. 10.1186/s12964-023-01351-6 37946175 PMC10633941

[B10] Kar A. Mukherjee S. Mukherjee S. Biswas A. (2024). Ubiquitin: A double-edged sword in hepatitis B virus-induced hepatocellular carcinoma. *Virology* 599:110199. 10.1016/j.virol.2024.110199 39116646

[B11] Kim J. Kwon H. Kalsoom F. Sajjad M. Lee H. Lim J. (2022). Ca2+/Calmodulin-dependent protein kinase II inhibits hepatitis B virus replication from cccDNA via AMPK activation and AKT/mTOR suppression. *Microorganisms* 10:98. 10.3390/microorganisms10030498 35336076 PMC8950817

[B12] Kim W. Nam S. Song H. Ko J. Park S. Kim H. (2012). The role of autophagy in unilateral ureteral obstruction rat model. *Nephrology* 17 148–159. 10.1111/j.1440-1797.2011.01541.x 22085202

[B13] Klieser E. Mayr C. Kiesslich T. Wissniowski T. Fazio P. Neureiter D. (2019). The crosstalk of miRNA and oxidative stress in the liver: From physiology to pathology and clinical implications. *Int. J. Mol. Sci.* 20:5266. 10.3390/ijms20215266 31652839 PMC6862076

[B14] Lee H. Cho Y. Lee G. You D. Yoo Y. Kim Y. J. (2018). A direct role for hepatitis B virus X protein in inducing mitochondrial membrane permeabilization. *J. Viral Hepat.* 25 412–420. 10.1111/jvh.12831 29193612 PMC7167162

[B15] Lin L. Lin Y. Han Z. Wang K. Zhou S. Wang Z. (2024). Understanding the molecular regulatory mechanisms of autophagy in lung disease pathogenesis. *Front. Immunol.* 15:1460023. 10.3389/fimmu.2024.1460023 39544928 PMC11560454

[B16] Lin Y. Zhao Z. Huang A. Lu M. (2020). Interplay between cellular autophagy and hepatitis b virus replication: A systematic review. *Cells* 9:2101. 10.3390/cells9092101 32942717 PMC7563265

[B17] Liu B. Fang M. He Z. Cui D. Jia S. Lin X. (2015). Hepatitis B virus stimulates G6PD expression through HBx-mediated Nrf2 activation. *Cell Death Dis.* 6:e1980. 10.1038/cddis.2015.322 26583321 PMC4670929

[B18] Liu S. Yao S. Yang H. Liu S. Wang Y. (2023). Autophagy: Regulator of cell death. *Cell Death Dis.* 14:648. 10.1038/s41419-023-06154-8 37794028 PMC10551038

[B19] Ma R. Li L. Yang H. Zou B. Ma R. Zhang Y. (2024). Therapeutic effect of nicotinamide mononucleotide on Alzheimer’s disease through activating autophagy and anti-oxidative stress. *Biomed. Pharmacother.* 178:117199. 10.1016/j.biopha.2024.117199 39053426

[B20] Miyakawa K. Nishi M. Ogawa M. Matsunaga S. Sugiyama M. Nishitsuji H. (2022). Galectin-9 restricts hepatitis B virus replication via p62/SQSTM1-mediated selective autophagy of viral core proteins. *Nat. Commun.* 13:531. 10.1038/s41467-022-28171-5 35087074 PMC8795376

[B21] Polaris Observatory Collaborators (2018). Global prevalence, treatment, and prevention of hepatitis B virus infection in 2016: A modelling study. *Lancet Gastroenterol. Hepatol.* 3 383–403. 10.1016/S2468-1253(18)30056-6 29599078

[B22] Popa G. Popa M. (2022). Oxidative stress in chronic hepatitis B-an update. *Microorganisms* 10:1265. 10.3390/microorganisms10071265 35888983 PMC9318593

[B23] Reina S. Guarino F. Magrì A. De Pinto V. (2016). VDAC3 As a potential marker of mitochondrial status is involved in cancer and pathology. *Front. Oncol.* 6:264. 10.3389/fonc.2016.00264 28066720 PMC5179545

[B24] Ren Q. Sun Q. Fu J. (2024). Dysfunction of autophagy in high-fat diet-induced non-alcoholic fatty liver disease. *Autophagy* 20 221–241. 10.1080/15548627.2023.2254191 37700498 PMC10813589

[B25] Sartorius K. Swadling L. An P. Makarova J. Winkler C. Chuturgoon A. (2020). The multiple roles of hepatitis B virus X protein (HBx) dysregulated MicroRNA in hepatitis B virus-associated hepatocellular carcinoma (HBV-HCC) and immune pathways. *Viruses* 12:746. 10.3390/v12070746 32664401 PMC7412373

[B26] Shen Y. He Y. Pan Y. Liu L. Liu Y. Jia J. (2024). Role and mechanisms of autophagy, ferroptosis, and pyroptosis in sepsis-induced acute lung injury. *Front. Pharmacol.* 15:1415145. 10.3389/fphar.2024.1415145 39161900 PMC11330786

[B27] Son J. Kim M. Lee J. Kim J. Chun E. Lee K. (2021). Hepatitis B virus X protein promotes liver cancer progression through autophagy induction in response to TLR4 stimulation. *Immune Netw.* 21:e37. 10.4110/in.2021.21.e37 34796041 PMC8568915

[B28] Thiruvengadam R. Venkidasamy B. Samynathan R. Govindasamy R. Thiruvengadam M. Kim J. (2023). Association of nanoparticles and Nrf2 with various oxidative stress-mediated diseases. *Chem. Biol. Interact.* 380:110535. 10.1016/j.cbi.2023.110535 37187268

[B29] Wang X. Lin Y. Liu S. Zhu Y. Lu K. Broering R. (2020). O-GlcNAcylation modulates HBV replication through regulating cellular autophagy at multiple levels. *FASEB J.* 34 14473–14489. 10.1096/fj.202001168RR 32892442

[B30] WHO (2021). *Global progress report on HIV, viral hepatitis and sexually transmitted infections, 2021. Accountability for the global health sector strategies 2016-2021: Actions for impact.* Geneva: World Health Organization.

[B31] Wing P. Liu P. Harris J. Magri A. Michler T. Zhuang X. (2021). Hypoxia inducible factors regulate hepatitis B virus replication by activating the basal core promoter. *J. Hepatol.* 75 64–73. 10.1016/j.jhep.2020.12.034 33516779 PMC8214165

[B32] Xie M. Yang Z. Liu Y. Zheng M. (2018). The role of HBV-induced autophagy in HBV replication and HBV related-HCC. *Life Sci.* 205 107–112. 10.1016/j.lfs.2018.04.051 29709654

[B33] Yamada T. Dawson T. Yanagawa T. Iijima M. Sesaki H. (2019). SQSTM1/p62 promotes mitochondrial ubiquitination independently of PINK1 and PRKN/parkin in mitophagy. *Autophagy* 15 2012–2018. 10.1080/15548627.2019.1643185 31339428 PMC6844492

[B34] Yoon K. Seo S. Lee K. Oh S. Park M. Hong S. (2023). Hepatitis B immunoglobulin inhibits the secretion of HBV via antigen-antibody precipitation in the multivesicular body. *Am. J. Transl. Res.* 15 5908–5920. 37854220 PMC10579012

[B35] Zhang L. (2020). Autophagy in hepatitis B or C virus infection: An incubator and a potential therapeutic target. *Life Sci.* 242:117206. 10.1016/j.lfs.2019.117206 31866520

[B36] Zhong L. Hu J. Shu W. Gao B. Xiong S. (2015). Epigallocatechin-3-gallate opposes HBV-induced incomplete autophagy by enhancing lysosomal acidification, which is unfavorable for HBV replication. *Cell Death Dis.* 6:e1770. 10.1038/cddis.2015.136 25996297 PMC4669713

[B37] Zhong L. Shu W. Dai W. Gao B. Xiong S. (2017). Reactive oxygen species-mediated c-Jun NH2-terminal kinase activation contributes to hepatitis B virus X protein-induced autophagy via regulation of the Beclin-1/Bcl-2 interaction. *J. Virol.* 91:e00001–e17. 10.1128/JVI.00001-17 28515304 PMC5512237

[B38] Zhou T. Jin M. Ding Y. Zhang Y. Sun Y. Huang S. (2016). Hepatitis B virus dampens autophagy maturation via negative regulation of Rab7 expression. *Biosci. Trends* 10 244–250. 10.5582/bst.2016.01049 27396843

